# Developing eHealth Interventions to Improve Diabetes Management in Emerging Adulthood: Qualitative Formative Study

**DOI:** 10.2196/75623

**Published:** 2025-11-27

**Authors:** April Idalski Carcone, Deepika Baskar, Aishah Mahmood, Karen MacDonell, Susan Eggly, Samiran Ghosh, Colleen Buggs-Saxton, Steven J Ondersma, Deborah A Ellis

**Affiliations:** 1 Department of Family Medicine and Public Health Sciences, School of Medicine, Wayne State University, 6135 Woodward Avenue, 3128 Integrative Biosciences Center, Detroit, MI, 48202, United States, 1 313-577-1057; 2 Department of Chronic Disease Epidemiology, School of Public Health, Yale University, New Haven, CT, United States; 3 Center for Translational Behavioral Science, Department of Behavioral Sciences and Social Medicine, Florida State University, Tallahassee, FL, United States; 4 Department of Oncology, School of Medicine, Wayne State University, Detroit, MI, United States; 5 Department of Biostatistics and Data Science, School of Public Health, The University of Texas Health Science Center at Houston, Houston, TX, United States; 6 Department of Pediatrics, School of Medicine, Wayne State University, Detroit, MI, United States; 7 Charles Stewart Mott Department of Public Health, College of Human Medicine, Michigan State University, Flint, MI, United States

**Keywords:** emerging adults, young adults, type 1 diabetes, behavioral intervention, eHealth

## Abstract

**Background:**

Emerging adulthood is a high-risk period during which many with type 1 diabetes demonstrate suboptimal diabetes management and glycemic control. There is a need for effective, scalable interventions designed specifically for this population. Technology-based approaches are readily accessible to this age group. Furthermore, interventions consistent with self-determination theory—which posits that the fulfillment of psychological needs for autonomy, self-efficacy, and relatedness promotes intrinsic motivation for change—may resonate well with emerging adults’ developmental needs for establishing independence and autonomy, and growing their social network.

**Objective:**

This study aimed to enhance the potential relevance, sustainability, and efficacy of 3 self-determination theory–informed mobile health intervention components and content for emerging adults with type 1 diabetes. Key areas of interest included emerging adults’ perspectives on the use of cultural tailoring, developmental relevance of content, and delivery preferences.

**Methods:**

In this qualitative formative study, 20 emerging adults reviewed and provided feedback on 3 newly developed intervention components via individual interviews. Ten reviewed the motivation enhancement system, a 2-session counseling intervention grounded in motivational interviewing and designed to enhance emerging adults’ autonomy and self-efficacy for diabetes self-management. Ten reviewed the SMS text messaging reminder intervention (one-way text message reminders to complete diabetes care) and the question prompt list (a list of questions related to diabetes care designed to increase patients’ active participation during medical visits). Interviews were analyzed using framework matrix analysis, an efficient approach to inductive thematic analysis.

**Results:**

Emerging adults found all 3 interventions acceptable and helpful. They noted the interventions’ *integration into the technology* they already use as a strength. Across interventions, emerging adults also expressed a preference for culturally tailored intervention content, including intervention examples, actors, and language representing their illness experience, identity, and personal preferences. Intervention-specific feedback suggested emerging adults liked motivation enhancement system intervention elements that were engaging (videos) and relatable (peer testimonials), and supported their growing autonomy and independence. For SMS text messaging reminders, emerging adults appreciated the straightforward nature of the reminders and recommended more directive messages. They appreciated the range of topics and variety of messages. Suggestions included making the messages more impactful (eg, direct, personalized, and engaging, such as using emojis). Emerging adults saw the question prompt list content areas as relevant and well-aligned with their concerns highlighting the topic of transitioning to adult life with diabetes as particularly salient.

**Conclusions:**

Emerging adult feedback supports the acceptability and use of these intervention components and will be used to refine the interventions. Feedback was especially positive regarding cultural and other tailoring efforts, as well as content directed at their pending transition to full independence. At the same time, their input suggests the need for multiple specific modifications, highlighting the importance of intensive and detailed feedback from end users.

## Introduction

Type 1 diabetes (T1D) management is complex, demanding, and primarily under the control of the individual [1]. Diabetes management often deteriorates during adolescence, affecting every component of diabetes care [2-5]. Once thought to be temporary, evidence suggests suboptimal diabetes management and glycemic control can persist into emerging adulthood [6,7]. Emerging adulthood spans adolescence and young adulthood (roughly aged 18‐25 y) and is characterized by growing independence, identity consolidation, and multiple life transitions [8]. Some have described emerging adulthood as the “most volatile years of life” [8].

Emerging adults self-report suboptimal diabetes management [5] demonstrating infrequent blood glucose monitoring (<1 test per day [9]) and suboptimal continuous glucose monitoring (CGM) wear rates [10]. Emerging adults have the worst glycemic health indicators of any age group [11], with hemoglobin A_1c_ (HbA_1c_) levels averaging from 8.4% to 9.3% [5,9] (levels <7% are recommended [12]); 83% fail to meet glycemic recommendations [9]. More concerning, by the age of 21 years, 32% of emerging adults have at least 1 diabetes-related complication or comorbidity [13]. Suboptimal diabetes management in emerging adults is related to decreased parental involvement in daily activities and increased reliance on peer and other support [8], transitioning from pediatric to adult clinical care, and infrequent clinic attendance [14].

While the challenges of managing T1D during emerging adulthood are well described [15-18], few efficacious interventions specifically designed for emerging adults with T1D have been developed. To date, the majority of emerging adult T1D intervention research has examined the use of advanced diabetes management technology (eg, CGM) [19-21] and closed-loop insulin delivery [22,23] or on optimizing the transition from the pediatric to the adult health care systems [24-26]. Other approaches are rare, with mixed results. In a pilot clinical trial of REAL-T, an occupational therapy intervention targeting diabetes management and its social ecology, the intervention improved glycemic control (HbA_1c_), diabetes-related quality of life, and blood glucose monitoring habits among emerging adults with T1D and type 2 diabetes. However, blood glucose monitoring and medication adherence behaviors were unchanged, as were mediators of behavior change (diabetes knowledge, problem-solving, and self-efficacy) [27]. Subsequently, a fully powered clinical trial of REAL-T for emerging adults with T1D, delivered via telehealth, showed improved diabetes-related quality of life and diabetes distress, but no change in glycemic outcomes, diabetes self-management, self-efficacy, or autonomy [28]. The Colorado Young Adults with T1D intervention combined telehealth clinical diabetes care with virtual peer support groups to improve diabetes outcomes (HbA_1c_, diabetes-related distress, depression, and self-efficacy) [29]. In a randomized controlled trial, per protocol analyses showed that participants in the active condition demonstrated significant improvement in glycemic control and CGM use, but depression symptoms increased, and diabetes distress and self-efficacy were unchanged. Intention-to-treat analyses were not published, and intervention participation rates were low, as 53% did not attend a single session. FAMS-T1D (Family and Friend Activation to Motivate Self-Care), a mobile health (mHealth) intervention to improve emerging adults’ self- and social-regulation, was tested in a pilot clinical trial (single group, and pretest or posttest design) and shown to be well-received (79% of those approached agreed to participate and 93% were retained). After intervention, emerging adults demonstrated improved diabetes management, self-efficacy, and diabetes distress, but changes in glycemic control were not statistically or clinically significant [30]. Other interventions for emerging adults demonstrate a similar pattern of either low engagement or limited improvement in glycemic outcomes [31-34].

Capitalizing on emerging adults’ unique developmental stage, in which feelings of autonomy and self-efficacy are paramount, may be key to intervention efficacy. Self-determination theory (SDT) [35] is an empirically derived theory of human motivation that posits three innate psychological needs: competence, relatedness, and autonomy. According to SDT, fulfilling these needs enhances intrinsic motivation and facilitates behavior change. To illustrate, autonomy-supportive communication by diabetes providers has been associated with better diabetes management in adolescents [36] and adults [37]. Because of their salient developmental need for independence, autonomy-focused interventions grounded in SDT may be particularly effective with emerging adults.

Unfortunately, there are few examples of autonomy-supportive interventions to improve diabetes outcomes in emerging adults with T1D. Guided self-determination (GSD) is an autonomy-oriented approach to shared decision-making involving the completion of 8 reflection worksheets over 8 to 12 months, which are discussed in 1-hour sessions with diabetes clinicians. While GSD increased adolescents’ perceptions of autonomy and decreased amotivation for diabetes self-management, it did not improve glycemic control [38]. Furthermore, 20% refused to participate, and 30% of those allocated to GSD did not complete the intervention. GSD for adults was reduced to 7 worksheets, or sessions, and clinical trial results indicated GSD improved diabetes distress, diabetes competence, amotivation, and perceptions of provider autonomy-supportive communication, but only improved glycemic control for women in the trial [39]. In addition, 25% of participants (age: mean 25.7, SD 5.1 y) either discontinued or failed to initiate treatment; completers required additional sessions (median 8; *R*=5‐14) to complete treatment. An online version of GSD for emerging adults improved autonomous motivation and perceptions of provider support for autonomy but did not impact glycemic control [31]. Online GSD was insufficient to improve engagement, as 65% of emerging adults failed to initiate treatment, only 28% of initiators completed treatment, and 66% were lost to follow-up at 6 months. Autonomy-supportive T1D camp (a 6-day experience intentionally designed to leverage SDT during diabetes education, meal planning assistance, camper-led parent training, glucose management, as well as typical camp activities) increased adolescents’ (aged 13‐18 y) sense of relatedness, but autonomy and competence remained unchanged; glycemic control was not examined [40]. Neither study examined the effect of autonomy-supportive interventions on diabetes self-management behavior.

This study was conducted in preparation for a larger clinical trial testing an autonomy-supportive intervention to improve diabetes self-management among older adolescents and emerging adults (aged 16‐25 y) with T1D and an elevated HbA_1c_ ≥9% [41]. The goal of this formative research was to enhance the potential relevance, sustainability, and efficacy of 3 SDT-informed mHealth intervention components to promote diabetes management and glycemic control and related intervention content for emerging adults with T1D by soliciting feedback from emerging adults with T1D and their recommendations for improving the interventions. Key areas of interest included emerging adults’ perspectives on the use of cultural tailoring, developmental relevance of content, and preferences for delivery logistics.

## Methods

### Design

Emerging adults with T1D participated in a single study visit during which they completed a brief demographic survey and reviewed either the motivation enhancement system (MES) intervention (10/20, 50%) or the text and question prompt list (QPL) interventions (10/20, 50%). Data were collected via a semistructured qualitative interview to obtain feedback on intervention content, delivery format, and suggestions for improvement.

### Participants

Emerging adults were recruited from a university-affiliated tertiary care outpatient diabetes clinic located in Detroit, Michigan, a large metropolitan city in the midwestern United States. Study inclusion criteria were individuals aged 16 to 25 years (inclusive) with a diagnosis of T1D for at least 6 months and HbA_1c_ greater than or equal to 9% currently and over the prior 6 months, English language fluency, and residence within 30 miles of the recruitment location. Exclusion criteria were determined via medical chart review and limited to medical diagnoses requiring atypical diabetes care or mental health conditions and developmental learning disabilities that resulted in the inability to independently participate in the study.

Potentially eligible emerging adults were identified via medical chart review. The initial data pull identified 701 potentially eligible emerging adults. Of these, 511 (73%) did not meet one or more study inclusion criteria. Research staff mailed a study introduction letter to 190 study-eligible emerging adults, or their caregivers when the emerging adults were aged <18 years. After a 2-week opt-out period, research staff followed up these letters with telephone calls to explain the details of the study, confirm study eligibility, and enroll interested, eligible emerging adults. Emerging adults were enrolled on a rolling basis until the target sample size of 10 emerging adults to review the MES intervention was achieved. This process was repeated for the review of the text and QPL interventions. A total of 20 emerging adults were enrolled, and 4 declined to participate.

### Procedures

Standardized procedures were used to minimize barriers to study participation and increase the integrity of the data collected. Study visits were completed using 2 strategies designed to overcome common barriers to participation (eg, lack of transportation). Initially, research staff conducted all study visits in emerging adults’ homes. Emerging adults used a study tablet to complete a brief demographic questionnaire via Qualtrics XM [42] and to review one or more of the intervention components. Research staff then engaged emerging adults in a semistructured qualitative interview. In March 2020, all study visits were transitioned from the home setting to online video conference calls due to the onset of the COVID-19 pandemic. The same procedures were followed, with minor modifications for the virtual context. Specifically, the consent or assent forms were emailed to study participants in advance of the study visit, and the study staff displayed them using the screen-sharing feature. Emerging adults completed the demographic questionnaire via a link sent via the videoconference software chat feature or verbally responded to the questions displayed via the screen-sharing feature. Similarly, research staff sent links to the online intervention components for emerging adults to review the intervention content, or research staff walked emerging adults through the intervention component using the screen-sharing feature.

### Interventions

All 3 interventions were developed and delivered using the Computerized Intervention Authorizing Software, version 3.0 (CIAS 3.0; Michigan State University) [43], a no-code Software-as-a-Service platform allowing development of interactive mobile web apps and SMS text messaging interventions without coding. The MES and QPL interventions were delivered via a link to a CIAS 3.0 intervention session, which will be texted to participants in the clinical trial. Both interventions leveraged CIAS 3.0’s interactive and emotive narrator. Consistent with human-computer interaction research [44], this narrator functions as an engaging guide throughout the intervention by reading text displayed on the screen and displaying emotional responses, such as surprise, sadness, or thoughtfulness, as appropriate. SMS text messaging was deployed by CIAS 3.0 as text messages. All 3 interventions were reviewed by a pediatric endocrinologist for clinical relevance prior to feasibility testing.

The MES is a brief (<15 min), 2-session, mHealth, counseling intervention grounded in motivational interviewing [45-48] and the Information-Motivation-Behavioral Skills model of health behavior change [49,50]. The Information-Motivation-Behavioral Skills model posits that behavior change results from the joint function of 3 critical components: accurate information about risk behaviors (eg, risks of suboptimal diabetes self-management) or their replacement behaviors (eg, benefits of effective diabetes self-management), motivation to change behavior, and behavioral skills necessary to perform the behavior (eg, self-efficacy). Motivational interviewing is an evidence-based strategy to optimize behavior change through the use of client-centered and goal-oriented directive communication to enhance intrinsic motivation and self-efficacy. MES content was developed to align with the needs and experiences of emerging adults. Specifically, we chose language emphasizing emerging adults’ diabetes self-management autonomy and curated content to be developmentally consistent with emerging adults’ experiences, including the reasons to engage in self-management activities, potential past successes, and personal strengths or weaknesses. Session 1 began with psychoeducation about the target behavior (ie, 3 key components of diabetes management referred to as “The 3Ms”: glucose monitoring, insulin administration [medicine], and dietary management [meals]) delivered via video clips from an endocrinologist and supported with a peer testimonial (emerging adults chose from 2 peer testimonials, one depicting a Black male and the other a Black female). Participants then advanced through motivation-enhancing exercises designed to increase feelings of autonomy and self-efficacy for changing the targeted illness behavior (ie, diabetes management). Session 1 ended with goal setting with three options: (1) completing the targeted illness behavior as recommended (ie, doing all diabetes care every day), (2) increasing the targeted illness behavior (ie, do more diabetes care every day), and (3) thinking more about changing one’s behavior, an autonomy-supportive option for those not yet ready to change their behavior. We then offered behavioral strategies to support goal attainment: setting reminders, establishing cues, and enlisting social support. Session 2 began by eliciting progress toward the diabetes care goal established in session 1 and then continued to grow motivation using motivation-enhancing exercises. In session 2, participants were asked which strategies they used and whether the strategies were helpful for completing diabetes care. Participants were then given the option to revisit the behavioral strategies information and reselect strategies to use.

The second intervention component was one-way tailored SMS text messages to remind emerging adults to complete their diabetes care. SMS text message reminders were grounded in social cognitive theory, which states that the individual, the behavior, and the environment reciprocally interact and influence one another [51]. Applied to diabetes self-management, SMS text message reminders promote self-management by providing an external prompt (environment) to the individual to complete the task. This process increases the likelihood of task completion, which leads to perceptions of control over the health behavior (autonomy), fosters feelings of competence (self-efficacy), and supports goal attainment [52-57]. SMS text message interventions may also generate feelings of social support [58], even when patients know the SMS text messages are automated [55]. SMS text messaging was adapted from a similar intervention to increase medication adherence for emerging adults with uncontrolled moderate-to-severe persistent asthma [59]. The asthma intervention consisted of 30 days of once-daily medication reminders via one-way SMS text message. In this study, we updated the content of the messages to reflect ‘The 3Ms” (ie, insulin administration, blood glucose testing, and carbohydrate counting). Texting was tailored to emerging adults’ preferences in two ways: emerging adults chose to receive reminders for one specific diabetes care component or a general reminder to complete “all their diabetes care,” and selected the time of day reminders were delivered.

The third intervention was a QPL, a communication tool to empower patients to actively participate during medical visits by asking questions and stating concerns and preferences [60,61]. QPLs are comprised of lists of questions related to the physical and psychosocial aspects of illness and illness treatment that patients may want to ask their physicians or other clinicians during a clinic visit. The theoretical foundation for the QPL resides in social cognitive theory, which posits that behavioral performance is largely a function of confidence in one’s ability to perform the behavior (self-efficacy) and the expectation that the behavior will result in the desired outcome [62]. Patients prepared with a QPL are more likely to ask questions and state their concerns, enabling shared decision-making and bolstering self-efficacy. The QPL was based on a similar tool designed to increase patients’ participation during oncology treatment interactions [63,64]. The QPL was developed by first compiling diabetes-related questions gleaned from a literature search, web-based resources, and consultation with 3 diabetes medical care providers. Questions addressed common concerns in the 3Ms’ domains and emerging adult-specific topics (eg, impact of tobacco or alcohol use on diabetes health). Intervention begins with a brief education component explaining the purpose of the QPL and the importance of communicating questions and concerns to their health care team. Emerging adults are then presented with lists of questions organized by content area from which they select the questions they would like to raise at the clinic visit. Emerging adults are routed to question sets tailored to their diabetes treatment regimen, for example, those monitoring their blood glucose with a glucometer are presented a set of questions specific to that method of blood glucose monitoring, and those using a continuous glucose monitor get a set of questions tailored to that approach. The questions selected by the emerging adults are compiled into a personalized report and emailed to the emerging adults upon completion and again the day prior to the clinic visit.

### Data Collection

The *Family Information Form* (*FIF*) is an investigator-developed measure to gather participant characteristics. The FIF collects participant demographic information such as age, race and ethnicity, sex assigned at birth, income, and family structure. The FIF also asks emerging adults for information about their illness, including the emerging adults’ date of diagnosis and current diabetes self-care regimen. Research staff used a *semistructured interview guide* developed for the study to guide the interviews. The interviews began with an explanation of the purpose of each intervention, after which the research staff guided emerging adults through a demonstration of the interventions. After reviewing the intervention, the research staff elicited emerging adults’ feedback on the intervention, including which elements emerging adults found most helpful and least helpful, suggestions for improving the intervention, and recommendations to make the interventions more consistent with the language and experiences of emerging adults (Multimedia Appendix 1). After 10 interviews, the feedback offered had reached the point of saturation and was deemed sufficient. Interviews were recorded and professionally transcribed for analysis.

### Data Analysis

A coding team composed of 2 coders (1 undergraduate psychology and 1 graduate public health student) and a qualitative expert (the principal investigator) analyzed the transcribed interview data using framework matrix analysis [65], an efficient approach to inductive thematic analysis. After an initial training period in which coders became familiar with the study design and aims, data collection procedures, and analytic approach, the coding team developed the initial coding matrix based on the content of the interview guide. Coders used this matrix to independently review the interview transcripts, “charting” summaries of emerging adults’ responses to interview questions into their coding matrix. Coders then met to compare their independently coded matrices and develop a consensus-coded matrix. Discrepancies were resolved through discussion and review of the interview transcripts. Once all interview transcripts were coded, the coders met to identify and summarize the themes (denoted with italicized text in the results) that emerged across emerging adults’ feedback. Coders developed descriptions of each theme with illustrative quotes extracted from the interview transcripts.

### Ethical Considerations

This study was approved by the Wayne State University institutional review board. Emerging adults provided informed consent or informed assent with parental informed consent to participate prior to engaging in study activities. Prior to analysis, all study data were deidentified to protect emerging adults’ privacy and confidentiality. Emerging adults were compensated with a US $50 gift card for their participation.

## Results

### Overview

Table 1 summarizes the characteristics of the emerging adults participating in this study. On average, emerging adults were 18.8 (SD 1.7) years, 50% (10/20) were female, and 80% (16/20) identified as Black, non-Hispanic. Emerging adults had completed an average of 12.0 (SD 1.8) years of education, reported their socioeconomic status as 5.9 (SD 1.6) out of 10 on the McArthur Scale of Subjective Socioeconomic Status [66], and most (13/20, 65%) were not employed. Emerging adults had been diagnosed with T1D for 11.1 (SD 4.8) years, 65% (13/20) used a glucometer to monitor their blood glucose levels, and 75% (15/20) administered their insulin via injection.

**Table 1.** Demographic and disease characteristics of study participants (N=20).

Characteristic	Value
Age (y), mean (SD)	18.8 (1.7)
Sex assigned at birth (female), n (%)	10 (50)
Race and ethnicity, n (%)	
Biracial, Hispanic	2 (10)
Black, non-Hispanic	16 (80)
White, Hispanic	2 (10)
Education (y), mean (SD)	12.0 (1.8)
Subjective socioeconomic status^a^, mean (SD)	5.9 (1.6)
Employment status, n (%)	
Employed part-time	5 (25)
Employed full-time	2 (10)
Not employed	13 (65)
Duration of diabetes (y), mean (SD)	11.1 (4.8)
Blood glucose monitoring method, n (%)	
Glucometer	13 (65)
Continuous glucose monitor	7 (35)
Insulin delivery method, n (%)	
Injected insulin	15 (75)
Insulin infusion pump	5 (25)

a The MacArthur Scale of Subjective Social Status uses a 10-point scale where 1=lowest social status and 10=highest social status

Emerging adults found all 3 interventions to be helpful and useful. They highlighted the interventions’ *integration into the technology* they use daily as a strength: “it has a high potential to be effective, especially with the amount of technology that’s used nowadays” and “I’m always on my phone as a young adult.”

### Motivation Enhancement System

Overall, emerging adults found the MES intervention *helpful* and the delivery structure *acceptable,* with 9 of 10 emerging adults reporting the length and number of sessions “About Right.” Emerging adults found the 3Ms mnemonic (3Ms=monitoring, medicine, and meals) a helpful prompt for remembering the essential diabetes care tasks:

*it would be something very helpful to remember because when you’re in the heat of the moment or you’re just not focusing, that’s something you should remember.*

When asked about the likelihood that emerging adults would recommend the MES program to other emerging adults, 8 of 10 emerging adults stated they would “certainly” recommend the program (mean 9.5/10, SD 1.1). When asked to elaborate, emerging adults highlighted the video elements of the intervention.

Emerging adults found the physician depicted in the educational video a *credible* and *trustworthy* authority figure:

*She got her message across, and it just makes sense. I understand why I need to do the things I need to do. The way she spoke about the things was very credible. I feel like I can trust her word.*

Emerging adults found the physician video content acknowledging the stress and challenge of diabetes care consistent with their own experiences:

*I had diabetes since I was four. So, when she [the physician] was like,* “*It begins to get hard,” and stuff like that, I understood that because it’s hard for me, especially now that I’m driving and working and doing other things.*

Emerging adults reported that the peer testimonial videos were *relatable* in two ways. First, the experiences depicted in the testimonial resonated with their own challenges with diabetes care:

*I can relate to [what] she’s saying too because I am tired too. It was way easier when I was a kid. Your parents may have to do all that, but now it’s my turn and it’s hard.*

Demographic similarities in the emerging adults depicted in the peer testimonial video enhanced participants’ feelings of relatability:

*She’s in college. She’s my age or around my age. Everything she was saying was the truth. Yeah, it was nice to see somebody who was my age similar to me. She’s same complexion. Yeah, it’s nice that there’s a black person talking to me.*

Emerging adults found other, nonvideo elements of the intervention credible and relatable, including lists of common barriers to diabetes care: “I’ve done almost everything on here.”

Emerging adults’ comments on the MES intervention highlighted its *autonomy-supportive* nature. Emerging adults reported less reliance on family support and input, and a desire for greater independence and control when making diabetes care decisions: “I don’t know if people would do something like that [enlist the support of family] in college.” Asking for help with diabetes care was viewed as undermining their autonomy: “that is one of my goals because I know how to ask for help, but I prefer not to.” Emerging adults explained that independently caring for their diabetes helped build their *self-efficacy* for diabetes care by giving them the sense that “I believe in myself” and that they were, therefore, in control of their health.

Emerging adults viewed the behavioral strategies to support diabetes care as helpful tools to help them be autonomous. To illustrate, one emerging adult found the suggestion to use reminders to prompt diabetes care useful:

*That’s smart, with the sticky note on the fridge or wherever they get their food from or try to get their food from. Just seeing that, it’s like,* “*all right, let me check my blood before I head out,” or* “*let me check my blood before I make this or eat this.” And for the putting an alarm on the phone, that’s very helpful, because when that time comes up and your alarm is screaming at you to do something, I feel like that’s very helpful as well.*

Another emerging adult found the suggestion to use cues to prompt diabetes care to be an effective strategy:

*It would be very much useful, especially for those who are always running around and forget to do things. Having those cues to put a thought in their head like,* “*wait, I should do this. I should do that before I do this or that.”*

A third emerging adult reported using a similar strategy to prompt blood glucose monitoring:

*I’ll put my [blood glucose monitoring] kit in the basket with my toothbrush and my soap and stuff. So, before I do any of that stuff, I got to pull this out and check my blood sugar. Or, if I have an early morning class, at night I’ll put this blood sugar kit in my jacket pocket. So, if I don’t check it when I wake up, by the time I get to the class or whatever, I’ll just pull it out and I’ll check it.*

### Text Message Reminders

Overall, emerging adults reported SMS text messaging would be a *helpful reminder* to stay “on track” with diabetes care tasks and increase the consistency of diabetes care:

*I’m trying to get on a schedule, but sometimes other things interfere with that. So, having those reminders will help.*

They thought receiving reminders at critical times would be particularly helpful, such as early morning (“when I wake up that’s the first notification I see on my phone”), afternoon (“the time where everybody would be up, so they will be alert when they get the message”), and late evening (“Sometimes you almost forget and it’s about to get late [around] bedtime”). Emerging adults *preferred SMS text messages* over email reminders because “a lot [of] people look at their phone more than they look at emails,” apps that “take up gigs [gigabytes/storage] on [my] phone,” and other technology-based reminders, which they tend to ignore:

*I already set up reminders in my phone. I don’t really pay attention to it though.*

Emerging adults’ *preferences for the content of SMS text message reminders* included “straightforward” wording of the SMS text messages and suggested making the language even more directive: “Take your insulin” versus “Don’t forget to take your insulin.” Emerging adults also preferred a variety of messages, suggesting we “use different ones, not the same wording every single day,” and a message personalized with their name. Using emojis in the messages was recommended to make them more engaging and consistent with other texts they receive: “Did you give insulin? with a smiley face.” Emerging adults emphasized the importance of being able to *tailor the reminders* to their own preferences. Specifically, the ability to select delivery times that aligned with their daily routines and mealtimes was a strength: “They’re very good choices because these are around [the] time you eat.” Most emerging adults thought a single reminder would be insufficient and recommended *multiple reminders per day*. While there was not strong agreement on the ideal number of texts, most emerging adults suggested 2 to 4 texts per day and advised that more than 7 texts would be viewed as “spamming.”

Finally, emerging adults recommended increasing the *range of topics* from which they might select reminder messages. Emerging adults suggested including reminders to exercise: “It’d probably motivate them or remind them that they maybe need to go out, take a jog, walk or lift some weights or just do some type of exercise to get their blood pumping.” Emerging adults recommended messages prompting blood glucose monitoring during exercise:

*I want to make sure that I’m not running low doing them [exercises], so having a reminder to make sure you’re on your sugar [checking glucose levels] while you’re doing exercise to make sure that it doesn’t run low or high.*

Emerging adults also suggested reminders to check ketones: “Make sure you check your ketones, if their reading’s above a certain level.”

### Question Prompt List

Overall, emerging adults reported that the QPL was a *useful tool to prepare for diabetes clinic visits*:

*It was incredibly useful. I think it would help me out at my next [doctor’s] appointment. It’s definitely less time consuming than sitting there researching stuff and writing it out on your own.*

The variety of questions was viewed as a strength:

*The questions were very diverse, very broad. There were some questions I had when I first got diabetes a long time ago versus some that I never thought to ask [my doctors] to this day.*

Emerging adults remarked that the QPL would be particularly useful for emerging adults newly diagnosed with diabetes:

*If I was older and I had gotten diagnosed, these would have been the kinds of questions I would ask.*

Emerging adults found the different *content areas of the QPL valuable*. They described the glucose monitoring questions as “beneficial,” “thoughtful,” and “diverse.” Emerging adults emphasized the importance of the insulin questions, noting the questions could help “prevent a situation” by elevating important discussion topics: “These ones are really important and specifically address things I may have overlooked.” Emerging adults who were administering their insulin via injection (8/10, 80%) thought the questions about transitioning to an insulin infusion pump were helpful: “I always wanted to know what is it or what does it do.” Those already using an insulin infusion pump (2/10, 20%) described these questions as very “relevant”: “All of these questions are questions that I had when I was transitioning to the pump.” One topic of particular interest to emerging adults was the section on transitioning to adult life with diabetes:

*I really like the adult life part because I think that [is] something that’s important that you consider before you get there rather than learn from experience.*

Emerging adults found these questions novel and thought-provoking, remarking:

*Some of the questions might change my mindset. Questions I didn’t know I had.*

They highlighted the importance of these questions for stimulating conversation on relevant but difficult-to-raise topics, including tattoos, pregnancy, and piercings: “I really think that these questions address some misconceptions that people may hear and feel nervous about.” Emerging adults' suggestions to expand the content of the QPL included adding questions about diabetes care challenges (“What are some of the struggles you are having with your diabetes?”) and to expand upon the diabetes care topics (“Why do I have a random high blood sugar if I haven’t eaten anything?”, “What type of insulin works better for me?”) and questions about “free carbs,” “insulin resistance,” and “how to navigate CGM sensor errors.”

When it came to esthetics and logistics, emerging adults expressed a desire to *tailor the intervention to their preferences*. Emerging adults stated they would prefer to rename the QPL but disagreed on what the preferred name should be. Preferences ranged from a highly personalized name integrating the patient’s own name into the title (“[Name]’s Diabetes Questions List”) to a “less diabetes specific” name (“Keeping My Life on Track”). Emerging adults also wanted to tailor how they received the finalized question list. Most emerging adults thought sending their diabetes question list directly to their doctor’s office would make it easier to communicate their concerns: “some people [are] not comfortable with their doctors yet or don’t like speaking out.” Some preferred receiving a copy of their question list by email, explaining they preferred email over SMS text message for a “bigger document” and “things that are important.” Some noted email delivery would allow them to “read it off of my phone,” noting “I usually always have my phone on me” and the accessibility of emails across devices: “You will always have it on file because it will be on email.” Emerging adults who preferred SMS text message delivery did so due to a personal preference (“I check my text messages more than I check my email”) and ease of access (“I have thousands of emails inside there, so I think text would be way better because I can just search it up”). One emerging adult suggested an option to mail the QPL for those without email access. Other tailoring preferences included font size, optional narration, delivery timing, and sharing their question lists with others.

## Discussion

### Principal Findings

The goal of this formative research was to enhance the potential relevance, sustainability, and efficacy of 3 SDT-informed mHealth intervention components to promote diabetes management and glycemic control and related intervention content for emerging adults with T1D by soliciting feedback from emerging adults with T1D and their recommendations for improving the interventions. Key areas of interest included emerging adults’ perspectives on the use of cultural tailoring, the developmental relevance of content, and preferences for delivery logistics. Overall, emerging adults found the interventions acceptable and thought they would be a helpful support for diabetes care. The integration of the interventions into technology that emerging adults already frequently use (mobile phone) was highlighted as a strength across interventions. Another common thread in emerging adults’ feedback was a preference for intervention content tailored to their illness experience, demographic characteristics, and personal preferences. Feedback related to the MES intervention highlighted elements that were engaging (videos), relatable (peer testimonials), and supportive of their growing autonomy and independence. Emerging adults viewed the text messaging intervention as covering a range of topics and had specific suggestions for making the messages more impactful (be direct, personalized, and engaging, eg, emojis). Emerging adults found the QPL content areas relevant and well-aligned with the concerns of emerging adults living with diabetes, particularly the topic of transitioning to adult life with diabetes.

It was unsurprising that emerging adults highlighted the interventions’ integration into mobile phone technology as a strength. Prior research has documented that adolescents and young adults prefer interventions delivered to their mobile phones [67], are more likely to use interventions when delivered to their personal device [68], and when delivered via text message [30,69]. However, to reap the benefits of eHealth interventions, engagement is critical [32,68]. Thus, finding ways to engage emerging adults in an intervention is a critical first step, and based on emerging adults’ feedback reported here, SMS text messaging–based interventions using emerging adults’ own devices may be an optimal strategy. The Unified Theory of Acceptance and Use of Technology [70] lends further support to this conclusion, as it suggests interventions integrated into the technology people use are more likely to be accepted and used because of the ease of use (effort expectancy). Other Unified Theory of Acceptance and Use of Technology factors that may be particularly relevant for emerging adults and SDT-based interventions are the perception that other people support using the intervention (social influence, given that peer influence is a critical factor for emerging adults [71]) and the automaticity of using the intervention (habit).

Emerging adults’ preference for intervention content tailored to their illness experience, demographic characteristics, and personal preferences is aligned with the broader literature on culturally tailoring interventions. Broadly speaking, cultural adaptation refers to the systematic modification of an intervention to align with the target audience’s cultural norms, beliefs, and values [72]. Culturally tailored interventions have demonstrated greater efficacy among both adult [73] and youth interventions [74]. Cultural tailoring for interventions targeting youth frequently includes adapting intervention methods (duration), language (translation), and content (relatable experiences) [74]. In this study, emerging adults highlighted several aspects of intervention tailoring. Emerging adults noted that the QPL questions aligned with questions they had previously asked during their own health care encounters or felt they would ask in future encounters. In the MES videos, emerging adults found the representation of peers with demographic characteristics (eg, age and race) similar to their own highly relatable. Others have noted the importance of representation of social identity as a key tailoring element for youth interventions [74]. In SMS text messaging, emerging adults viewed the ability to tailor the timing and content of reminders to personal preferences as a strength. In light of this feedback, we increased the planned once-daily reminder to twice daily based on emerging adults’ feedback reported here. Most emerging adults’ recommendations for intervention improvement related to augmenting the capacity to tailor the interventions to personal preferences, including increasing the frequency and duration of intervention, augmenting the intervention content, and personalizing reminders with the recipient’s name. Tailoring to individual preferences is consistent with emerging adults’ developmental need for autonomy and has been noted as an important element for engagement in technology-based interventions [75,76].

The interventions developed in this study were all grounded in SDT [35]. SDT was selected as the guiding framework because of the resonance-enhancing feelings of autonomy, self-efficacy, and relatedness it has for emerging adults who are at a developmental stage in which forging independence, a unique identity, and social connections are paramount [8]. Emerging adults participating in this study noted that each intervention contained elements that resonated with this framework. Specifically, emerging adults found the credibility and trustworthiness of the videos embedded in the MES intervention promoted autonomous diabetes care. The videos’ relatability, particularly the peer testimonial, helped foster feelings of relatedness. Emerging adults found other MES elements, such as the behavioral strategies to support diabetes care goals, also fostered feelings of autonomy. Emerging adults embraced text messaging as a strategy to promote consistent diabetes care, an approach previously linked to improvements in diabetes self-efficacy [55]. Emerging adults noted that multiple QPL questions were questions they had in the past or would consider asking in the future, fostering both feelings of confidence (self-efficacy) and relatedness. Questions addressing adult issues (eg, alcohol use) resonated with emerging adults’ goal of independence (autonomy). While theory-based interventions are more likely to improve glycemic control and psychological outcomes [77], SDT-guided interventions, to date, have not demonstrated improvements in glycemic control [38,40]. GSD, the most widely tested SDT intervention, relies upon the completion of worksheets that form the basis of conversations with diabetes educators [31,37,39]. Not only has this approach failed to demonstrate improvements in glycemic control, but poor recruitment and retention rates also suggest this approach is unacceptable to emerging adults [31,38,39].

### Limitations

This study’s small sample size limits the generalizability of this study to other emerging adults and populations with T1D. This limitation is offset by the fact that the emerging adults included were mostly non-Hispanic Black and were selected based on suboptimal glycemic control from an urban setting. Such populations are underrepresented in research literature and frequently absent from intervention development research. In addition, the findings reported here parallel those from a similar study conducted with a mainly White (89%) rural population of emerging adults who reviewed the same interventions and also highlighted the excellent fit of technology-delivered interventions for their age group, expressed a preference for individualized interventions, and stated the value of intervention elements that enhanced their feelings of autonomy [78]. While the inclusion of historically marginalized emerging adults is a strength, other cultural factors, eg, emerging adults’ acceptance of their diabetes, may have influenced emerging adults’ feedback and intervention preferences [79].

A second limitation was constraining emerging adults’ engagement in the research to providing feedback and recommendations on our investigator-developed intervention components. A more inclusive approach would have been to engage emerging adults in the development of the interventions using a community-based participatory approach. However, as described in Multimedia Appendix 1, the interventions adapted in this study were derived from similar interventions developed and empirically tested in similar populations. Furthermore, each intervention component is theoretically grounded in SDT and related behavior change frameworks. Finally, emerging adults’ feedback will be used to refine the interventions prior to a planned randomized controlled trial using the multiphase optimization strategy [80] to test the intervention components’ preliminary efficacy to improve glycemic control and build the most efficacious multicomponent intervention [41]. Specifically, MES will be refined based on emerging adults’ language preferences. The planned frequency of SMS text messaging will be increased from 1 to 2 messages per day, with the number of messages increased to minimize repetition and redundancy. SMS text messaging will also be further tailored to include the emerging adult’s name in messages. QPL questions will be revised according to emerging adults’ preferences and new questions added to augment the scope of question content. Emerging adults’ feedback will also help other researchers develop interventions aligned with the needs and preferences of emerging adults, which will increase their acceptability and future effectiveness.

Finally, the development and use of digital interventions assumes the end users, in this instance, emerging adults with T1D, have the necessary digital literacy to effectively engage with the intervention and that they have access to the tools (digital devices and internet access) necessary to receive the intervention. In the United States, internet use and smartphone ownership are at an all-time high. The most recent statistics from the Pew Research Center indicate 95% of US adults use the internet, 90% have a smartphone, and 80% subscribe to high-speed internet at home [81]. Smartphone ownership is even greater for young adults aged 18 to 29 years (97%), and 78% subscribe to high-speed internet at home [81]. Today’s adolescents and young adults are growing up immersed in technology (cell phones and computers), making technology-based interventions a natural extension of their social ecology [82-84]. Thus, mHealth interventions exclude only a minority of youth and emerging adults who are “digital natives” [85] having grown up with technology as a component of their everyday life.

### Conclusions

The results reported here provide clinicians and researchers with specific guidance for effective behavioral interventions with emerging adults with T1D. First, clinicians and researchers working with emerging adults should offer eHealth interventions that leverage the technology emerging adults already use, namely, their mobile phones. They should also provide opportunities for emerging adults to tailor interventions to their personal preferences, such as providing options for the frequency and duration of intervention, and the specific content of the intervention, for example, selecting a discrete behavioral target from a menu of options. Clinicians and researchers should also ensure interventions match the cultural context of emerging adults, such as ensuring case examples, vignettes, and audio-visual content represent people of a similar background and with similar experiences, to increase engagement, a critical factor for sustained use and to reap the benefits of an intervention. Finally, interventions grounded in SDT align well with emerging adults’ innate psychological needs for autonomy, self-efficacy, and relatedness. Thus, interventions that activate these psychological processes may be particularly effective for evoking behavior change among emerging adults.

## Supplementary material

10.2196/75623 Multimedia Appendix 1 Semistructured interview questions and detailed descriptions of the interventions.

## Abbreviations

CGM: continuous glucose monitoring
CIAS 3.0: Computerized Intervention Authorizing Software, version 3.0
FAMS-T1D: Family and Friend Activation to Motivate Self-Care
FIF: Family Information Form
GSD: guided self-determination
HbA_1c_: hemoglobin A_1c_
MES: motivation enhancement system
mHealth: mobile health
QPL: question prompt list
SDT: self-determination theory
T1D: type 1 diabetes
