# Supplementary material for: Developing eHealth Interventions to Improve Diabetes Management in Emerging Adulthood: Qualitative Formative Study
Source: JMIR Form Res. 2025 Nov 27;9:e75623. doi: 10.2196/75623 (PMC12661604; doi:10.2196/75623)
Supplement: Multimedia Appendix 1 [file formative-v9-e75623-s001.docx]

Supplemental Materials

**Supplement 1: Semi-Structured Interview Questions**

1. Motivation Enhancement System (MES)
   1. Session 1
2. I’d like to start by hearing, in your own words, what you thought of the program. What did you think?
3. Which parts of the session did you find the most helpful?
4. Which parts of the program did you find the least helpful?
5. What parts of the program should we definitely keep?
6. If you could change any part of the program, what would you change?
7. What parts of the session would you cut out altogether?
8. Now, I’d like to ask you about some more specific aspects of the program. During the program, you had the opportunity to view two videos. In the first video, Dr. Anderson talked about the benefits of completing all your diabetes care every day. What did you think about Dr. Anderson’s video?
9. Now, I’d like to show you two versions of the doctor video. You just saw two versions of a doctor talking about diabetes. First, which version did you prefer, the first or the second?
10. Some people prefer the first/second video because the doctor is more directive: she tells patients what they should do to take care of their diabetes. Did you notice this?
11. In the second/first video, the doctor takes a different approach; she encourages patients but explains that it is their choice to change how they take care of their diabetes. Did you notice this?
12. Now that we’ve talked about the differences between the videos, do you still prefer? Do you think most youth would prefer a directive approach or a non-directive approach? Why?
13. Dr. Anderson introduced “The 3 Ms” of diabetes care. They were monitoring your blood glucose throughout the day, taking all your insulin every day, or medicine, and counting the carbohydrates you eat at every meal and snack. What did you think about “The 3Ms”?
14. For the second video, you may have had the option of hearing from either Gabrielle or Michael about their experience taking care of diabetes. If you viewed the video, did you choose Gabrielle or Michael. What did you think of this video?
15. Now, I’d like to talk with you about some of the other elements that were part of the program. As you made your way through the program there were a number of lists from which you could choose ideas or experiences related to diabetes care. For example, the first list was common barriers to competing diabetes care. Barriers are things that could get in the way of doing diabetes care. Depending on the choices you made throughout the program, you might not have seen all of these lists, but I’d still like to hear your thoughts about these lists, even if you didn’t see them as you went through the program. Let’s start with the list of common barriers. What do you think of this list?
16. The second list describes benefits, or “good” things that might happen if people with diabetes consistently complete all their daily diabetes care tasks. What do you think of this list?
17. This one lists a number of personal strengths. Personal strengths are qualities that people have within themselves that might help them get through a difficult time or problem. What do you think of these personal strengths?
18. Finally, there was a list of past successes. This list contained a number of different experiences that people might have been successful at in the past. When I say past successes, I mean accomplishments that you or someone like you may have experienced. What do you think of these experiences?
    1. MES Session 2
19. I’d like to start by hearing what you thought of Session 2?
20. Which parts of Session 2 did you find the most helpful?
21. Which parts of Session 2 did you find the least helpful?
22. If you could change any part of Session 2, what would you change?
23. If parts of Session 2 were changed, what should we keep?
24. What parts of Session 2 would you cut out altogether?
25. Now, I’d like to get your thoughts on the treatment program as a whole. On a scale of 1-10, with 1 being not at all helpful and 10 being extremely helpful, how helpful do you think this program would be in helping you complete your diabetes care every day? Why did you rate the program a {refer to the numerical rating}?
26. What do you think about the number of sessions? Would you say there are too many sessions, too few, or the right number?
27. What do you think about the length of the program sessions? Would you say that the sessions are too long, too short, or the right length?
28. On a scale of 1-10, with 1 being not at all likely and 10 being definitely, how likely would you be to recommend the program to someone else living with diabetes?
29. What other thoughts do you have about the program?
30. Text Message Reminders (TXT)
31. What do you think of the text message reminder program?
32. If you could change any part of the program, what would you change?
33. How do you think the text message reminder program would change your diabetes care?
34. Now, I’d like to talk with you about some specific parts of the program. Let’s start with the types of diabetes self-management (self-care) covered by the text messages. People will be able to choose to receive text message reminders about blood sugar monitoring, taking insulin, counting carbs, or all three of these diabetes care tasks. Please take a minute to look over the sample text message reminders. When you’re ready, we can start with any thoughts that you might have about these areas of diabetes self-management.
35. What do you think about the wording of the text messages?
36. People in the text messaging part of the program will get one text message reminder each day. What do you think about the frequency of one text message per day?
37. People in the text messaging part of the program will be asked what time of day they would like to get their text message reminder. Their choices will be early morning, mid-morning, around noon, afternoon, evening, late evening, night, and late-night. The specific times are on this slide. What do you think about these choices?
38. What other thoughts do you have about the text message reminder part of the program?
39. Question Prompt List (QPL)
40. I’d like to start by hearing, in your own words, what you thought of the program. What did you think?
41. If you could change any part of the program, what would you change?
42. If we were going to change the program, what should we definitely keep?
43. Now, I’d like to talk with you about some of the other elements that were part of the program. Let’s start with the name of the question list which is “My Diabetes Question List”. What do you think of this name?
44. Next, I’d like to take a closer look at the different questions on the question list. Let’s start with the blood glucose monitoring questions. How are you monitoring your blood sugar right now?
45. On the next three slides are the questions about monitoring with a blood glucose meter. Please take a minute to look over these questions. When you’re ready, we can start with any thoughts that you might have about these questions.
46. The next two slides have questions about monitoring with a continuous glucose monitor. Please take a minute to look over these questions. When you’re ready, we can start with any thoughts that you might have about these questions.
47. We suggested two reasons people might not be monitoring their blood sugar: not having the supplies they need and feeling tired or burnt out when it comes to diabetes. What do you think of these reasons for not monitoring?
48. When you are monitoring your blood glucose, how do monitor, with a meter or CGM?
49. These next three slides are about monitoring with a blood glucose meter. Please take a minute to look over these questions. When you’re ready, we can start with any thoughts that you might have about these questions.
50. These next two slides are about monitoring with a continuous glucose monitor. Please take a minute to look over these questions. When you’re ready, we can start with any thoughts that you might have about these questions.
51. I’d like to talk about insulin next. These next two slides have questions about insulin. Please take a minute to look over them. When you’re ready, we can start with any thoughts that you might have about these questions.
52. How are you getting your insulin right now?
53. This slide has questions that people who are interested in knowing more about an insulin pump might ask. Please take a minute to look over this them. When you’re ready, we can start with any thoughts that you might have about these questions.
54. This slide has questions that are specific to using an insulin pump. Please take a minute to look over this list. When you’re ready, we can start with any thoughts that you might have about these questions.
55. This slide has questions deal with diet and exercise. Please take a minute to look over them. When you’re ready, we can start with any thoughts that you might have about these questions.
56. The final set of questions on the next two slides deal with other concerns people like you living with type 1 diabetes might want to talk with their medical team about. Please take a minute to look over them. When you’re ready, we can start with any thoughts that you might have about these questions.
57. At the end of the program, you will be sent a copy of your diabetes question list by email. What do you think of this list?
58. How would you like to get your diabetes question list? By text? By email? Some other way?
59. We plan to send the diabetes question list right after it’s completed and again the day before a diabetes appointment. What do you think of this plan?
60. What other thoughts do you have about the diabetes question list?

**Supplement 2: Detailed Descriptions of the Interventions**

***Motivation Enhancement System (MES)***

The Motivation Enhancement System (MES) is a brief (<15 minute), two session, mHealth, counseling intervention grounded in Motivational Interviewing (MI[1-4]) and the Information-Motivation-Behavioral Skills (IMB) model of health behavior change.[5, 6] The IMB model posits that behavior change results from the joint function of three critical components: accurate information about risk behaviors (e.g., risks of suboptimal diabetes self-management) or their replacement behaviors (e.g., benefits of effective diabetes self-management), motivation to change behavior, and behavioral skills necessary to perform the behavior (e.g., self-efficacy). MI is an evidence-based strategy to optimize behavior change through use of client-centered and goal-oriented directive communication to enhance intrinsic motivation and self-efficacy. The MES intervention was originally developed to reduce drug use among urban, post-partum women.[7, 8] Our research group has successfully adapted MES to improve self-management for persons living with a variety of chronic illnesses including EAs with HIV who are newly prescribed antiretroviral medication,[9, 10] EAs with uncontrolled moderate to severe persistent asthma,[11, 12] and preadolescents with T1D.[13-15]

For the present study, we adapted the T1D MES content to align with the needs and experiences of EAs. Specifically, we chose language emphasizing EAs’ diabetes self-management autonomy and curated content to be developmentally consistent with EA experiences, including the reasons to engage in self-management activities, potential past successes, and personal strengths/weaknesses. As outlined in Figure 1 below, session 1 began with psychoeducation about the target behavior, i.e., three key components of diabetes management referred to as ‘The 3Ms’: glucose **m**onitoring, insulin administration (**m**edicine), and dietary management (**m**eals) which was delivered via video clips from an endocrinologist and supported with a peer testimonial. We updated the peer testimonial video to be delivered by EAs. We developed two peer testimonials, one depicting a Black male and the other a Black female, and EAs were given the option to choose which testimonial to hear as they progressed through the intervention. Participants then advanced through motivation-enhancing exercises designed to increase feelings of motivation and self-efficacy for changing the targeted illness behavior (i.e., diabetes management). Session 1 ended with goal setting with three options: (1) completing the targeted illness behavior as recommended (i.e., doing all diabetes care every day), (2) increasing the targeted illness behavior (i.e., do more diabetes care every day), and (3) thinking more about changing one’s behavior, an autonomy-supportive option for those not yet ready to change their behavior. After goal selection, we added a new intervention content area, behavioral strategies to support goal attainment. The behavioral strategies included setting reminders, establishing cues, and enlisting social support.

Session 2 began by eliciting progress toward the diabetes care goal established in Session 1 and then continued to grow motivation using motivation-enhancing exercises. Participants were asked which of the behavioral strategies introduced in Session 1 they used and whether the strategies were helpful for completing diabetes care. Participants were then given the option to revisit the behavioral strategies information and re-select strategies to use. EAs provided feedback on the adapted MES via qualitative interviews.


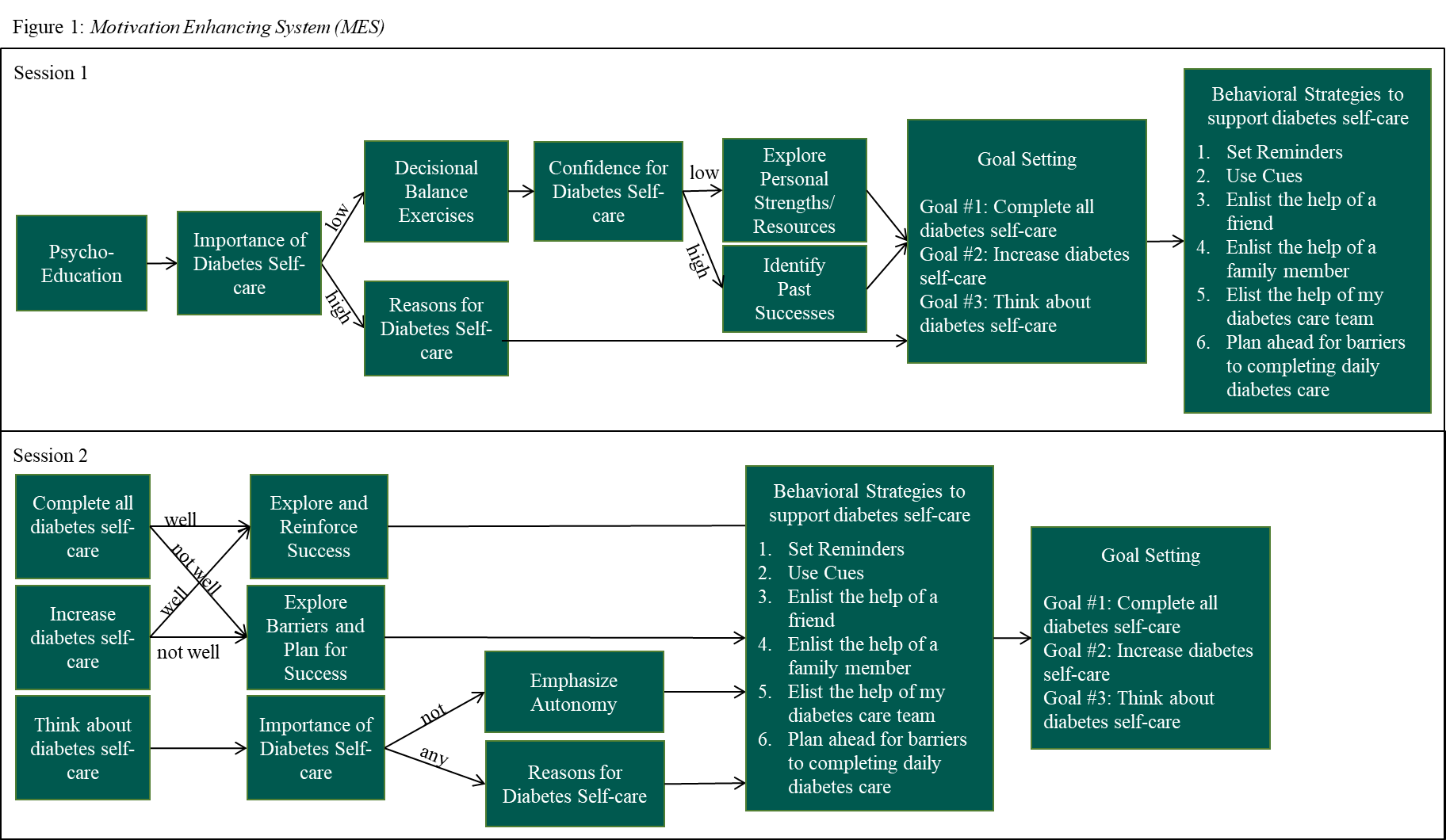


***Text Message Reminders (TXT)***

The Text Message Reminders intervention (TXT) was one-way tailored text messages to remind EAs to complete their diabetes care. Text message reminders were grounded in social cognitive theory which states the individual, the behavior, and the environment reciprocally interact and influence one another.[16] Applied to diabetes self-management, text message reminders promote self-management by providing an external prompt (environment) to the individual to complete the task. This process increases the likelihood of task completion which leads to perceptions of control over the health behavior (autonomy), fosters feelings of competence (self-efficacy) and supports goal attainment.[17-22] Text message interventions have also been shown to generate feelings of social support,[23] even when patients know the text messages are automated.[20]

The TXT intervention tested in this study was adapted from a similar intervention to increase medication adherence for EAs with uncontrolled moderate-to-severe persistent asthma.[12] The asthma intervention consisted of 30 days of once daily medication reminders via one-way text message. Prior to the formative interviews with EAs, we updated the content of the messages to reflect the key components of the T1D regimen (insulin administration, blood glucose testing, and carbohydrate counting). EAs could choose to receive reminders for one of these components or a general reminder to “complete all their diabetes care”. EAs were also able to tailor the timing of reminder delivery. Figure 2 illustrates TXT for EAs with T1D.


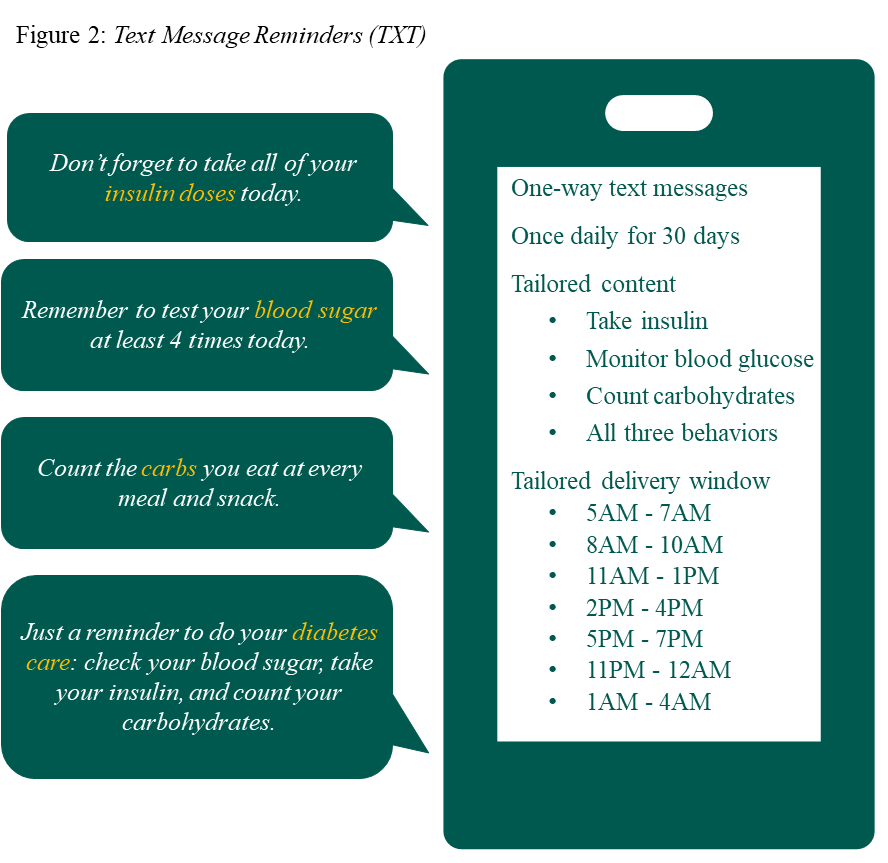


***Question Prompt List (QPL)***

The Question Prompt List intervention (QPL) was a communication tool to empower patients to actively participate during medical visits, for example, by asking questions and stating concerns and preferences.[24, 25] QPLs are comprised of lists of questions related to the physical and psychosocial aspects of illness and treatment that patients may want to ask their physicians or other clinicians during a clinic visit. The theoretical foundation for the QPL resides in social cognitive theory which posits that behavioral performance is largely a function of confidence in one’s ability to perform the behavior (self-efficacy) and the expectation that the behavior will result in the desired outcome.[26] Patients prepared with a QPL are more likely to ask questions and state their concerns, enabling shared decision making and bolstering self-efficacy. The QPL developed for this study was based on a cancer QPL designed to increase patients’ participation during oncology treatment interactions. [27, 28]

The diabetes QPL was developed by first compiling diabetes-related questions gleaned from a literature search, web-based resources, and consultation with three diabetes medical care providers. Questions addressed common concerns in the three diabetes care domains (blood glucose monitoring, insulin administration, and dietary management) and EA-specific topics (e.g., impact of tobacco or alcohol use on diabetes health). The intervention begins with a brief education component explaining the purpose of the QPL and the importance of communicating questions and concerns to their health care team. EAs are then presented lists of questions organized by content area from which they select the questions they would like to raise at the clinic visit. EAs are routed to question sets tailored to their diabetes treatment regimen, for example, those monitoring their blood glucose with a glucometer are presented a set of questions specific to that method of blood glucose monitoring and those using a continuous glucose monitor get a set of questions tailored to that approach. The questions selected by the EA are compiled into a personalized report and emailed to the EA upon completion and again the day prior to the clinic visit. Figure 3 illustrates the intervention flow of the QPL for EAs with T1D.


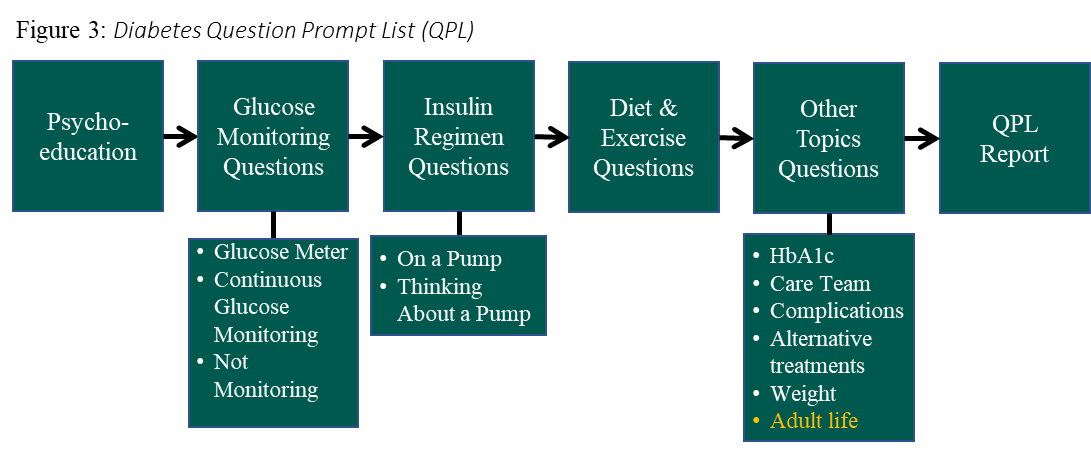


**References**

1. Miller WR, Rollnick S. Motivational interviewing: Preparing people to change addictive behavior. New York, NY, US: The Guilford Press; 1991. xvii, 348 p. ISBN: 0-89862-566-1 (hardcover).

2. Miller WR, Rollnick S. Motivational Interviewing: Preparing People for Change. Second ed. New York: Guilford; 2002.

3. Miller WR, Rollnick S. Motivational Interviewing: Helping people change. Third ed. New York: The Guilford Press; 2012 September 7, 2012. 482 p. ISBN: ISBN-10: 1609182278

ISBN-13: 978-1609182274.

4. Miller WR, Rollnick S. Motivational interviewing: Helping people change and grow. New York: Guilford Publications; 2023. ISBN: 1462552811.

5. Fisher JD, Fisher WA. Changing AIDS-risk behavior. Psychological Bulletin. 1992;111(3):455-74. doi: 10.1037/0033-2909.111.3.455.

6. Fisher WA, Fisher JD, Harman J. The Information-Motivation-Behavioral Skills Model: A general social psychological approach to understanding and promoting health behavior. Jerry Suls KAW, editor2003. 82-106 p.

7. Ondersma SJ, Chase SK, Svikis DS, Schuster CR. Computer-based brief motivational intervention for perinatal drug use. Journal of Substance Abuse Treatment. 2005;28(4):305-12. doi: 10.1016/j.jsat.2005.02.004.

8. Ondersma SJ, Svikis DS, Schuster CR. Computer-based brief intervention: A randomized trial with postpartum women. American Journal of Preventive Medicine. 2007;32(3):231-8. doi: 10.1016/j.amepre.2006.11.003.

9. Naar-King S, Outlaw AY, Sarr M, Parsons JT, Belzer M, MacDonell K, et al. Motivational Enhancement System for Adherence (MESA): Pilot Randomized Trial of a Brief Computer-Delivered Prevention Intervention for Youth Initiating Antiretroviral Treatment. Journal of Pediatric Psychology. 2013;38(6):638-48. doi: 10.1093/jpepsy/jss132.

10. Outlaw AY, Naar-King S, Tanney M, Belzer M, Aagenes A, Parsons JT, et al. The Initial Feasibility of a Computer-Based Motivational Intervention for Adherence for Youth Newly Recommended to Start Antiretroviral Treatment. Aids Care. 2014 Jan 2;26(1):130-5. PMID: WOS:000328728100017. doi: 10.1080/09540121.2013.813624.

11. MacDonell K, Gibson-Scipio WM, Lam P, Naar-King S, Secord E, editors. The Detroit Young Adult Asthma Project: Feasibility and Acceptability of a Multi-Component, Technology-Based Intervention Targeting Adherence to Controller Medication. American Thoracic Society International Conference Abstracts; 2015: American Thoracic Society.

12. MacDonell KK, Naar S, Gibson-Scipio W, Lam P, Secord E. The Detroit Young Adult Asthma Project: Pilot of a Technology-Based Medication Adherence Intervention for African-American Emerging Adults. Journal of Adolescent Health. 2016;59(4):465-71.

13. Idalski Carcone A, Ellis DA, Naar S, Ondersma SJ, Moltz K, Dekelbab B, et al. Enhancing Parental Motivation to Monitor African American Adolescents’ Diabetes Care: Development and Beta Test of a Brief Computer-Delivered Intervention. JMIR research protocols. 2014;3(3):e43. PMID: 25236503. doi: 10.2196/resprot.3220.

14. Rajkumar D, Ellis DA, May DK, Carcone A, Naar-King S, Ondersma S, et al. Computerized Intervention to Increase Motivation for Diabetes Self-Management in Adolescents with Type 1 Diabetes. Health Psychol Behav Med. 2015;3(1):236-50. PMID: 26973800. doi: 10.1080/21642850.2015.1079716.

15. Ellis DA, Carcone AI, Ondersma SJ, Naar-King S, Dekelbab B, Moltz KC. Brief Computer-Delivered Intervention to Increase Parental Monitoring in Families of African American Adolescents with Type 1 Diabetes: A Randomized Controlled Trial. Telemedicine and e-Health. 2017;23(6):493-502. doi: 10.1089/tmj.2016.0182.

16. Bandura A. Social Cognitive Theory. Six Theories of Child Development. Greenwich, CT: JAI Press; 1989. p. 1-60.

17. Herbert L, Owen V, Pascarella L, Streisand R. Text message interventions for children and adolescents with type 1 diabetes: a systematic review. Diabetes Technol Ther. 2013 May;15(5):362-70. PMID: 23550554. doi: 10.1089/dia.2012.0291.

18. Arora S, Peters AL, Agy C, Menchine M. A mobile health intervention for inner city patients with poorly controlled diabetes: proof-of-concept of the TExT-MED program. Diabetes Technology & Therapeutics. 2012;14(6):492-6. doi: 10.1089/dia.2011.0252. PMID: 22524591.

19. Dick JJ, Nundy S, Solomon MC, Bishop KN, Chin MH, Peek ME. Feasibility and usability of a text message-based program for diabetes self-management in an urban African-American population. Journal of Diabetes Science and Technology. 2011 Sep 1;5(5):1246-54. PMID: 22027326. doi: 10.1177/193229681100500534. PMID: 22027326.

20. Nundy S, Dick JJ, Solomon MC, Peek ME. Developing a behavioral model for mobile phone-based diabetes interventions. Patient Educ Couns. 2013 Jan;90(1):125-32. PMID: 23063349. doi: 10.1016/j.pec.2012.09.008.

21. Nundy S, Mishra A, Hogan P, Lee SM, Solomon MC, Peek ME. How do mobile phone diabetes programs drive behavior change? Evidence from a mixed methods observational cohort study. Diabetes Educator. 2014 Nov-Dec;40(6):806-19. PMID: 25278512. doi: 10.1177/0145721714551992.

22. Franklin VL, Waller A, Pagliari C, Greene SA. A randomized controlled trial of Sweet Talk, a text-messaging system to support young people with diabetes. Diabet Med. 2006 Dec;23(12):1332-8. PMID: 17116184. doi: 10.1111/j.1464-5491.2006.01989.x.

23. Fischer HH, Moore SL, Ginosar D, Davidson AJ, Rice-Peterson CM, Durfee MJ, et al. Care by cell phone: text messaging for chronic disease management. The American Journal of Managed Care. 2012;18(2):e42-7.

24. Brandes K, Linn AJ, Butow PN, van Weert JC. The characteristics and effectiveness of Question Prompt List interventions in oncology: a systematic review of the literature. Psychooncology. 2015 Mar;24(3):245-52. PMID: 25082386. doi: 10.1002/pon.3637.

25. Sansoni JE, Grootemaat P, Duncan C. Question Prompt Lists in health consultations: A review. Patient Educ Couns. 2015 Jun 3. PMID: 26104993. doi: 10.1016/j.pec.2015.05.015.

26. Street Jr RL, Slee C, Kalauokalani DK, Dean DE, Tancredi DJ, Kravitz RL. Improving physician–patient communication about cancer pain with a tailored education-coaching intervention. Patient education and counseling. 2010;80(1):42-7.

27. Eggly S, Tkatch R, Penner LA, Mabunda L, Hudson J, Chapman R, et al. Development of a question prompt list as a communication intervention to reduce racial disparities in cancer treatment. Journal of Cancer Education. 2013;28(2):282-9.

28. Eggly S, Hamel LM, Foster TS, Albrecht TL, Chapman R, Harper FWK, et al. Randomized trial of a question prompt list to increase patient active participation during interactions with black patients and their oncologists. Patient Education and Counseling. 2017;100(5):818-26. doi: 10.1016/j.pec.2016.12.026.
